# STRScan: targeted profiling of short tandem repeats in whole-genome sequencing data

**DOI:** 10.1186/s12859-017-1800-z

**Published:** 2017-10-03

**Authors:** Haixu Tang, Etienne Nzabarushimana

**Affiliations:** School of Informatics and Computing, Indiana University, 150 S. Woodlawn Avenue, Bloomington, 47405 IN USA

**Keywords:** Short tandem repeats, Whole-genome sequencing, Algorithm, DNA forensics

## Abstract

**Background:**

Short tandem repeats (STRs) are found in many prokaryotic and eukaryotic genomes, and are commonly used as genetic markers, in particular for identity and parental testing in DNA forensics. The unstable expansion of some STRs was associated with various genetic disorders (e.g., the Huntington disease), and thus was used in genetic testing for screening individuals at high risk. Traditional STR analyses were based on the PCR amplification of STR loci followed by gel electrophoresis. With the availability of massive whole genome sequencing data, it becomes practical to mine STR profiles *in silico* from genome sequences. Software tools such as lobSTR and STR-FM have been developed to address these demands, which are, however, built upon whole genome reads mapping tools, and thus may not be sensitive enough.

**Results:**

In this paper, we present a standalone software tool STRScan that uses a greedy algorithm for targeted STR profiling in next-generation sequencing (NGS) data. STRScan was tested on the whole genome sequencing data from Venter genome sequencing and 1000 Genomes Project. The results showed that STRScan can profile 20% more STRs in the target set that are missed by lobSTR.

**Conclusion:**

STRScan is particularly useful for the NGS-based targeted STR profiling, e.g., in genetic and human identity testing. STRScan is available as open-source software at http://darwin.informatics.indiana.edu/str/.

## Background

Short tandem repeats (STRs), also referred to as the microsatellites or simple-sequence repeats (SSRs), are a short stretch of DNA containing approximately two to 30 tandemly repeated units of 1–6 bps. STRs are present in many prokaryotic and eukaryotic genomes, including mammalian genomes such as human [1, 2]. Over half a million STRs are characterized in human genome, composing approximately 3% of the entire human genome [3]. Due to their high polymorphism, STRs are commonly used as genetic markers [4–7]. In particular, a small set of STR loci can be used for identity and parental testing ([8, 9]), in which multiple STR loci were amplified by using PCR in a small amount of human DNA from one (sometimes unknown) source and the length of PCR products are compared against one or more human DNA samples from the other sources (e.g., in a forensic database). This *STR typing* procedure has been largely standardized, and the putative STR loci subject to such test were collected in public database such as STRBase [10].

Although STRs are largely considered as “junk DNA”, some STRs locate in protein coding genes, whose products were shown to play functional roles in higher organisms, e.g., the glutamine-rich domains participating in transcription regulation [11]. Even the STRs in non-coding regions may be involved in the expression regulation of their downstream genes [12]. In particular, the unstable expansion of trinucleotide repeats were known to be associated with human diseases [13]. A preeminent example is the Huntington disease, a genetic neurodegenerative disorder caused by the expansion of a tandem repeat of CAG triplet in the Huntington (*HTT*) gene, resulting in a different protein form that may lead to brain degeneration [14]. As such, STR profiling in disease susceptible alleles is often used as a genetic testing tool for individuals at high risk of inheriting these genetic disorders [15].

The traditional experimental analysis of STRs involved the amplification of the target STR locus by PCR, using unique sequences in the flanking regions of the STR as primers, followed by the length measurement of the PCR product using gel electrophoresis, which indicates the copy number of the target STR. In recent years, whole genome sequencing (WGS) becomes more affordable owning to the rapid advance in next-generation sequencing (NGS) techniques. Conventional software tools such as tandem repeat finder (TRF) [16] can detect novel STRs from assembled genome sequence, such as the human genome [17]. New software tools and pipelines such as lobSTR [18] and STR-FM [19] have also been developed that can be directly applied for the STR profiling in WGS data. The power of the STR analysis from NGS data has been demonstrated in a recent study, which showed that the surname of a human individual can be inferred from the personal genome sequencing data through the analysis of the profiles of Y chromosome STRs (Y-STRs) and online genealogy database [20]. The genome-wide STR profiling tools have enabled the survey of STR variations in human population [19, 21, 22]. It was also shown that a substantial number of STR loci are pervasively expressed in human population, which may represent a novel set of regulatory variants in the human genome ([23]).

In this paper, we present a standalone software tool STRScan for the profiling of STRs in next-generation sequencing (NGS) data. Here, we adopted a targeted approach to STR profiling: instead of mining all STRs at a whole genome scale (as the goal of lobSTR or STR-FM), we attempted to study only a user-defined subset of STR loci, a scenario particularly useful for forensic or genetic testing [24], and thus avoid the time-consuming genome-wide reads mapping procedure. As a result, our method is not limited by the sequence comparison against the STR loci represented as linear DNA sequences in a reference genome, and thus can adopt a fine-tuned alignment algorithm for STR identification in DNA sequences. In addition to mining whole-genome sequencing data, our method can be applied directly to STR profiling in NGS data from targeted STR samples, after STR enrichment [25], or PCR amplification of specific set of STR loci (e.g., for identity or genetic testing).

In STRScan, each STR locus is represented by a pattern including the tandem copies of one or more repetitive units, along with the upstream, downstream and the intermediate sequences between repetitive units, which can be constructed from the reference genome sequence of an organism (e.g., human), and the occurrence of each STR locus in a sequence read is identified by using a greedy seed-extension strategy. Because our goal is to profile STRs from population sequencing data (e.g., the 1000 genome sequencing data), we assume the difference between the STR pattern and its occurrence in the sequence reads are caused by single nucleotide polymorphisms (SNPs) or sequencing errors, and thus composes only a small fraction of the entire locus. Therefore, STRScan used the edit distance to measure the difference between a STR pattern and its occurrence in a read, and only reports those occurrences below a small threshold (i.e., *δ*).

We tested STRScan on the whole genome sequencing (WGS) data from both the Sanger sequencer [26] and the Illumina sequencer (generated by the 1000 Genomes Project [27]). Comparing with existing software tools like lobSTR and STR-FM, STRScan can identify significantly (in average 20%) more STRs from NGS data, while using comparable or less computation time. Hence, STRScan is ready to be used for targeted profiling of STRs in sequencing data and for STR typing through DNA amplification followed by next-generation sequencing.

## Methods

**Fig. 1:**
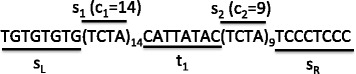
A schematic illustration of the pattern of a STR locus consisting of two tandem repeating units of four base-pairs long each

A locus of short tandem repeat (STR) is defined as a sequence of *n* short repeats, each consisting of a *repetitive unit* repeating multiple times, and spacing sequences between every two consecutive short repeats. Formally, a STR locus is represented as a pattern $\phantom {\dot {i}\!}P=s_{L}(s_{i})_{c_{i}}t_{i}s_{R}$, in which *s*_*i*_ and *c*_*i*_ (*i*=1,2,...,*n*) represent the DNA string and the copy number of the *i*-th repetitive unit, respectively, *t*_*i*_ represents the intermediate string between the *i*-th and the (*i*+1)-th repetitive units (and thus *t*_*n*_=*∅*), and *s*_*L*_ and *s*_*R*_ represent the unique strings at the upstream or downstream spanning the entire STR locus (Fig. 1). Given a DNA string *Q* and a STR pattern *P*, their distance *D*(*Q*,*P*)computed along an optimal alignment between them, which can be viewed as the concatenation of the alignment between each component of *P* and their counterpart in *Q*. Specifically, let (*q*_*L*_,*q*_1_,*p*_1_,...,*q*_*n*_,*p*_*n*_,*q*_*R*_) be a partition of the sequence *Q*, (i.e., *Q* is the concatenation of the substrings: *Q*=*q*_*L*_·*q*_1_·*p*_1_·...·*q*_*n*_·*p*_*n*_·*q*_*R*_), the distance between *Q* and *P* for this specific partition is defined as $D_{(q_{L}, q_{i}, p_{i}, q_{R})}(Q, P) = D(q_{L}, s_{L}) + \sum ^{n}_{i=1} [D((q_{i})^{m}, s_{i}) + D(p_{i}, t_{i})] + D(q_{R}, s_{R})$, where *D*(*q*,*s*) is the minimum distance (e.g., the edit distance or its variants) between the strings *s* and *q*, and (*q*_*i*_)^*m*^ is a tandem repeat of *q*_*i*_ in *m* copies (|*m*−*n*|≤*ε*, where *ε* is the maximum variants of the *i*-th repetitive unit) that has the smallest distance with *s*_*i*_. For each short read *T* in a given NGS dataset, our objective of STR profiling is to find if there exists a subsequence *t* of *T*, such that the minimum distance between *t* and *P*, *D*(*t*,*P*) is below a given threshold *δ*.

We used a greedy seed-extension strategy to address the STR profiling problem. We assume the difference between the STR pattern and its occurrence is so small that the occurrence contains a substring of length *k* that is the exact tandem copy of one repetitive unit in the STR pattern. As a result, we can index the STR patterns based on the seeds representing the tandem repeats of *k* bases long. For example, if a STR pattern contains a repetitive unit *s*_*i*_=*ATCC* with *c*_*i*_=8 copies, the pattern can be indexed by the seed of *ATCCATCCATCCATCCATCC* for *k*=20. Note that if *k* is not a multiple of the repetitive unit length, we can truncate the last copy of the repetitive unit in the tandem repeat: in the example above, for *k*=18, the seed becomes *ATCCATCCATCCATCCAT*. Furthermore, we also assume we can use the *fitting alignment* algorithm to find a substring *t* ^′^ in *T* with the smallest distance with a string *s*. In practice, we compute the edit distance between two strings using a banded dynamic programming algorithm [28] that constrains the total number of indels to be no more than a small band *ω*.

Built upon these two components, the STRScan algorithm takes as input a set of STR patterns and a set of NGS reads, and identifies each sequencing read containing a substring that matches one STR locus (i.e., with edit distance below *δ*). The algorithm consists of three steps: 1) the input set of STR patterns are indexed by *k*-mers of tandem repeats in the STR loci; 2) the *k*-mers in each read is searched against the indexed *k*-mers from the STR patterns, and the matched *k*-mers are represented as the *seed* alignments between corresponding reads and STR patterns; and 3) each seed alignment will be extended by using the fitting alignment algorithm. Specifically, assuming that a seed alignment between the STR pattern *P* and the read *T* with the distance D(P, T) containing *m* copies of the *i*-th repetitive unit (*s*_*i*_) in *P* and its 3’-end is aligned with the *j*-th nucleotide in *T* (if the last repetitive unit in the *k*-mer is truncated, we first extend the seed alignment to the end of the repetitive unit by using gap-free extension), we consider the possible extensions of the seed alignment with the minimum distance:

1 $$ {}D'(P,T)\! =\! D(P,T) + min\! \left\{\! \begin{array}{ll} D(s_{i}, T^{*}_{j+1}), & \text{if }{m < n + \epsilon}, \\ D(t_{i} \cdot s_{i+1}, T^{*}_{j+1}), & \text{if }{i < n}, \\ D(s_{R}, T^{*}_{j+1}), & \text{if } i = n. \end{array}, \right.  $$

where $T^{*}_{j}$ represents the suffix of *T* starting at the *j*-th position, and *s*·*t* represents the concatenation of the two strings *s* and *t*. The alignment extension with the minimum distance is then appended into the current seed alignment, and the distance score and the end position in *T* are updated accordingly. The procedure is iterated until the alignment reaches the downstream sequence (*s*_*R*_) or the distance becomes above the threshold of *δ*. A similar extension algorithm can be applied to the 5’-end of the seed alignment simultaneously until it reaches the upstream sequence *s*_*L*_,

2 $$ {}D'(P,T)\! =\! D(P,T) + min\! \left\{\! \begin{array}{ll} D(s_{i}, T'_{k-1}), & \text{if }{m < n + \epsilon}, \\ D(t_{i} \cdot s_{i-1}, T'_{k-1}) & \text{if }{i > 1}, \\ D(s_{L}, T'_{k-1}) & \text{if }{i = 1} \end{array}\!, \right.  $$

where *k* represents the first position in *T* at the 5’-end of the seed alignment, and $T^{\prime }_{k}$ represents the prefix of *T* ending at the *k*-th position.

## Results

We tested STRScan on three whole genome sequencing (WGS) datasets: one obtained by using Sanger sequencers [26], whereas the other two obtained by using Illumina sequencers [27]. The first dataset (denoted as the *Venter* dataset) was downloaded from NCBI Trace Archive, consisting of about 12.5 millions of reads of 1000 bps. The other two datasets (denoted by their individual IDs, HG00145 and HG00140, respectively) were selected from the 1000 Genomes project, and downloaded from the Short Read Archive (Project ID: SRR099957 for HG00145, and ERR251013 for HG00140), consisting of 115.5 and 65.8 millions of read pairs, respectively, with each read of 100 bps long. In each of these datasets, we attempted to search for reads supporting the STRs from two different panels, which are commonly used in DNA forensics: the YSTR penal consisting of 18 STRs from human Y chromosome, and the Combined DNA Index System (CODIS) panel consisting of 14 STRs from autosomes [29]. The copy number of the repeating unit in each identified targeted STR was reported by STRScan along with the supporting reads. When two or more different copy numbers are observed in the supporting reads, the corresponding STR is classified as *multi-allelic*: for Y chromosome STRs, the multiple alleles are likely located in different locus of Y chromosome, whereas for CODIS STRs, the multiple alleles may reflect the heterozygosity of the STR in the personal genome.

We compared the performance of STRScan and lobSTR [18] on three sets of testing data. As shown in Table 1, STRScan identified 31 reads in the Venter dataset, supporting a total of 15 out of 18 STRs in the Y chromosome STR panel, whereas lobSTR identified 20 reads supporting a total of 11 STRs. STRScan identified all STR alleles reported by lobSTR, and four additional STRs with valid supporting reads (see Supplementary website http://darwin.informatics.indiana.edu/str/ for the sequences of the supporting reads). The copy numbers reported by STRScan are in agreement with the result of lobSTR on the 11 STRs identified by both methods. Similarly, STRScan identified 34 supporting reads in the Venter dataset, supporting 12 out of 14 STRs in the CODIS panel, which contains all 9 STRs identified by lobSTR (supported by 21 reads). STRScan also outperforms lobSTR on identification of STRs in short reads obtained by using Illumina sequencers. For the two testing datasets from 1000 Genome project. For example, in the HG00140 dataset, STRScan identified 10 reads supporting 7 STRs in the Y chromosome STR panel, whereas lobSTR identified 5 reads supporting 4 STRs, and STRScan identified 12 reads supporting 7 STRs in the CODIS panel, whereas lobSTR identified 7 reads supporting 6 STRs. Similar results were obtained in the HG00145 dataset (see Table 1). Overall, STRScan identified 31 reads supporting STRs in these two datasets, whereas lobSTR identified 19 reads, with 11 reads in common.

**Table 1:** Comparison of STRScan and lobSTR on STR identification from shotgun sequencing reads

STR markers	Chromosome / location	# in reference genome	Copy number of identified STRs (number of supporting reads)					
			Venter		HG00145		HG00140	
			STRScan	lobSTR	STRScan	lobSTR	STRScan	lobSTR
YSTR (on Y chromosome) panel								
DYS19	chrY 9521989-9522052	15	14(1)	-	-	-	-	-
DYS385^a^	chrY 20801599-20801642	11	11(2)	11(1)	11(3)	-	12(1)	-
	chrY 20842518-20842573	14	14(1)	14(1)				
DYS388	chrY 14747535-14747570	12	12(2)	12(1)	-	-	-	-
DYS389I	chrY 14612242-14612289	12	13(3)	13(1)	-	-	-	-
DYS389II	chrY 14612242-14612405	29	29(2)	29(2)	-	-	-	-
DYS390	chrY 17274947-17275042	24	23(1)	23(1)	15(1)	-	-	-
DYS391	chrY 14102795-14102838	11	10(1)	10(1)	-	-	10(2)	10(2)
DYS392	chrY 22633873-22633911	13	13(2)	13(2)	-	-		
DYS393	chrY 3131152-3131199	12	13(2)	-	-	-	-	-
DYS426	chrY 19134850-19134885	12	12(1)	12(1)	-	-	-	-
DYS437	chrY 14466994-14467057	16	-	-	-	-	16(2)	-
DYS438	chrY 14937824-14937873	10	12(1)	12(1)	-	-	10(1)	10(1)
DYS439	chrY 14515312-14515363	13	12(1)	12(1)	-	-	11(1)	11(1)
DYS447	chrY 15278740-15278854	23	25(1)	-	-	-	-	-
DYS448	chrY 24365070-24365225	19	-	-	-	-	-	8(1)
DYS460 (A7.1)	chrY 21050842-21050881	10	12(2)	-	-	-	11(1)	-
H4	chrY 18743553-18743600	12	-	-	12(1)	12(1)	11(2)	-
YCAII^a^	chrY 19622111-19622156	23, 23	19(3), 23(5)	19(3), 23(4)	19(1)	19(2)	-	-
	Total	18	15(31)	11(20)	4(6)	2(3)	7(10)	4(5)
CODIS (on autosomes) panel								
CSF1PO	chr5 149455887-149455938	13	11(7)	11(5)	-	-	11(1)	11(1)
D13S317^a^	chr13 82722160-82722203	11	12(1),13(2)	11(1)	-	-	-	-
D16S539	chr16 86386308-86386351	11	12(2)	-	13(1)	-	11(2)	11(1)
D18S51	chr18 60948900-60948971	18	14(2)	14(2)	-	-	15(1)	-
D21S11	chr21 20554291-20554417	29	-	-	-	-	-	-
D3S1358^a^	chr3 45582231-45582294	16	16(3)	16(3)	-	-	-	-
D5S818	chr5 123111250-123111293	11	-	-	-	-	-	-
D7S820	chr7 83789542-83789593	13	10(3)	10(2)	-	-	8(3)	-
D8S1179	chr8 125907107-125907158	13	12(1)	12(1)	8(1)	6(2)	-	13(1)
FGA^a^	chr4 155508888-155508975	22	26(1), 21(1)	26(1), 21(1)	-	-	-	-
PentaD	chr21 45056086-45056150	13	13(2)	-	9(1)	9(1)	-	-
PentaE	chr15 97374245-97374269	5	12(2)	12(1)	-	-	13(1)	13(1)
TH01	chr11 2192318-2192345	7	6(2)	-	-	-	5(1),10(2)	10(2)
TPOX	chr2 1493425-1493456	8	8(5)	8(4)	-	-	8(1)	8(1)
	Total	14	12(34)	9(21)	3(3)	2(4)	7(12)	6(7)

^a^Multi-allelic STR markers, each with two alleles on the reference human genome

## Discussion

Our results showed that short reads obtained from conventional next-generation sequencing techniques (e.g., Illumina sequencers for whole genome sequencing) may not be suitable for targeted profiling of STRs: only a small number of reads can be identified supporting common STR panels (such as Y Chromosome and CODIS) in whole genome sequencing data. On the other hand, relatively longer reads from Illumina miSeq, which may reach the length of 500–600 bps, comparable to the length of Sanger sequencing reads as in Venter genome datasets, are much more sensitive for targeted STR profiling (as shown in Table 1). When combined with targeted amplification of specific STR loci, miSeq sequencing may achieve satisfactory sensitivity for STR typing in DNA forensics and for targeted STR profiling in genetic disease screening. In the future, we plan to test the performance of STRScan on more forensic sequencing datasets when they become publicly available.

## Conclusion

In this paper, we present STRScan, which allows the targeted search of an user-defined panel of short tandem repeats (STRs) in whole-genome sequencing data. Comparing to existing tools (such as lobSRT) designed for blind genome-wide mining, STRScan showed improved sensitivity on identifying sequencing reads supporting STRs with various copy numbers at specific loci, as it employs a fast greedy algorithm to compare the read sequence and putative STRs.

## Abbreviations

CODIS: Combined DNA Index System
NGS: Next-generation sequencing
PCR: Polymerase chain reaction
SNP: Single-nucleotide polymorphsim
SSR: Simple-sequence repeats
STR: Short tandem repeats
WGS: Whole-genome sequencing
